# Identifying Myoclonic Epilepsy Misdiagnosed as Psychogenic Nonepileptic Seizures: Challenges in Differential Diagnosis

**DOI:** 10.7759/cureus.62653

**Published:** 2024-06-18

**Authors:** Ateeba Ahmed, Pradeep S Patil

**Affiliations:** 1 Psychiatry, Jawaharlal Nehru Medical College, Datta Meghe Institute of Higher Education and Research, Wardha, IND

**Keywords:** treatment, seizure disorders, diagnosis, eeg monitoring, myoclonic epilepsy, psychogenic nonepileptic seizures (pnes)

## Abstract

Psychogenic nonepileptic seizures (PNES) and epileptic seizures often present with similar clinical manifestations. This case report describes the diagnostic journey of a 24-year-old female initially diagnosed with PNES but later found to have myoclonic epilepsy upon comprehensive evaluation. The patient presented with recurrent episodes characterized by sudden loss of awareness, jerking movements, and urinary incontinence, often triggered by stressors. Initial assessment, including video-electroencephalography (EEG) monitoring, did not reveal epileptiform activity, leading to the provisional diagnosis of PNES. However, the persistence of symptoms and doubts regarding the diagnosis prompted further investigation, which uncovered generalized spike-and-wave discharges on repeat EEG studies. The diagnosis of myoclonic epilepsy was established based on these findings, and treatment with valproate resulted in a significant reduction in seizure frequency. This case underscores the importance of a thorough evaluation in distinguishing between seizure disorders and psychogenic manifestations, emphasizing the need for collaborations between neurology and psychology disciplines for accurate diagnosis and management.

## Introduction

Psychogenic nonepileptic seizures (PNES) and epileptic seizures often present with similar clinical manifestations, posing a diagnostic challenge for clinicians. PNES, previously referred to as pseudo-seizures, are paroxysmal events resembling epileptic seizures but lack electrographic evidence of epileptic activity [1]. These events are considered to be of psychological origin, typically associated with underlying emotional stressors or trauma [2]. Epilepsy, on the other hand, is a chronic neurological disorder characterized by recurrent unprovoked seizures resulting from abnormal neuronal activity in the brain [3]. It encompasses a diverse spectrum of seizure types, including generalized tonic-clonic seizures, absence seizures, and myoclonic seizures, among others. Accurate diagnosis and classification of seizures are essential for appropriate treatment selection and prognosis [4].

Differentiating between PNES and epileptic seizures requires a comprehensive evaluation integrating clinical history, semiology, electroencephalography (EEG), and neuroimaging findings [5]. While EEG is a valuable tool in the diagnosis of epilepsy, it may not always capture the epileptiform activity during seizures, especially in cases of focal seizures or those with nonconvulsive status epilepticus [6]. Moreover, the coexistence of psychological factors in patients with epilepsy further complicates the diagnostic process. Patients with epilepsy may experience psychosocial stressors, psychiatric comorbidities, or psychogenic episodes, which can influence the clinical presentation and complicate the interpretation of diagnostic tests [7].

In recent years, advances in neuroimaging techniques, genetic testing, and biomarker research have provided additional insights into the underlying mechanisms of seizure disorders, facilitating more accurate diagnosis and personalized treatment approaches [8]. Additionally, a multidisciplinary collaboration involving neurologists, epileptologists, neuropsychologists, and psychiatrists is crucial in the holistic management of patients with seizures, addressing both the neurological and psychological aspects of their condition [9]. Therefore, in this case report, we aimed to differentiate between these two conditions.

## Case presentation

A 24-year-old female presented to our neurology clinic with a history of recurrent episodes that had been ongoing for approximately six months. She described these episodes as sudden, brief loss of awareness accompanied by jerking movements of her limbs and trunk. Additionally, she reported urinary incontinence during these episodes. The events typically lasted a few seconds to a minute and were followed by confusion and fatigue. There was no reported aura preceding the episodes, and she denied experiencing any focal neurological deficits between the events. Upon further inquiry, the patient reported that the episodes occurred irregularly, with no discernible pattern. However, she noted that they seemed more frequent during increased stress or emotional distress. She reported experiencing significant life stressors in the months leading up to the onset of these episodes but denied any recent head trauma, febrile illness, or substance use.

The patient had no past medical history, including no history of epilepsy, head injuries, or psychiatric disorders. Her family history was unremarkable for epilepsy or other neurological conditions. She was not taking any regular medications and had no known drug allergies. On physical examination, the patient appeared well-nourished and in no acute distress. Vital signs were within normal limits, and no focal neurological deficits were noted. The initial neurological examination, including cranial nerve examination and motor, sensory, and coordination assessments, was unremarkable.

Given the clinical presentation suggestive of seizures, further investigations were pursued to establish a diagnosis. EEG monitoring was performed during one of the episodes. However, no epileptiform activity was detected on EEG during the observed event, leading to a provisional diagnosis of PNES. A psychological assessment was also undertaken to explore potential underlying psychological factors contributing to the presentation. The patient was found to have unresolved emotional conflicts and significant stressors in her personal life, raising suspicion of a psychogenic etiology for the episodes.

Despite the provisional diagnosis of PNES, doubts regarding the diagnosis persisted due to the discrepancy between the clinical presentation and the absence of epileptiform activity on EEG. Repeat EEG studies were performed during wakefulness and sleep, revealing generalized spike-and-wave discharges, predominantly occurring in the morning.

**Figure 1:**
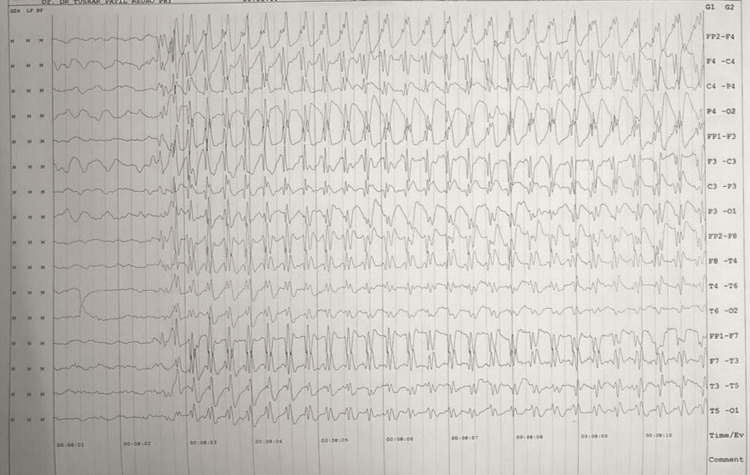
An electroencephalogram showing abnormalities

**Figure 2:**
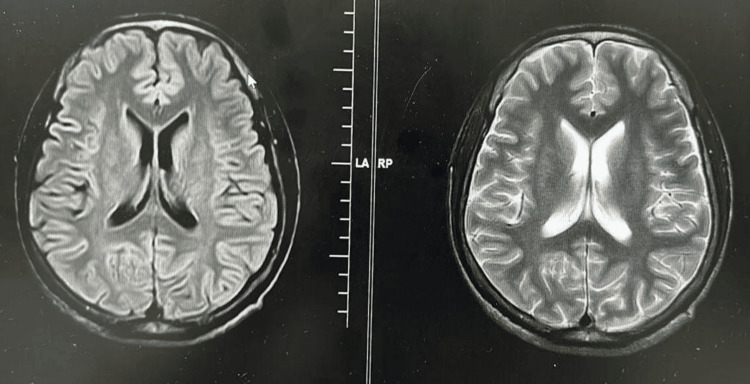
MRI of the brain showing no abnormalities

Considering the EEG findings suggestive of epileptic activity, coupled with the persistence of symptoms despite psychogenic interventions, a diagnosis of myoclonic epilepsy was entertained. Figure 1 shows the EEG findings. Brain magnetic resonance imaging (MRI) was unremarkable, ruling out structural abnormalities (Figure 2). The patient was initiated on anti-epileptic medication (valproate), resulting in a significant reduction in the frequency of her episodes. The patient was scheduled for regular follow-up appointments to monitor treatment response and adjust medication dosages as necessary. Additionally, she was referred to a psychologist for ongoing psychotherapy to address the underlying psychological stressors contributing to her condition.

## Discussion

The case presented here underscores the complexities involved in diagnosing and managing seizure disorders, particularly in cases where the clinical presentation overlaps with psychogenic nonepileptic seizures. PNES pose a diagnostic challenge due to their clinical resemblance to epileptic seizures, often necessitating a multidisciplinary approach for accurate diagnosis and appropriate management. The initial assessment of the patient's episodes, including EEG monitoring during one of the events, did not reveal epileptiform activity, leading to the provisional diagnosis of PNES. This finding is consistent with the previous literature highlighting the difficulty in diagnosing PNES solely based on clinical presentation and EEG findings [10]. PNES are considered a diagnosis of exclusion, and their accurate diagnosis relies on the absence of epileptiform activity during events captured on EEG [11].

However, the persistence of symptoms despite psychogenic interventions prompted further investigation in our case. Repeat EEG studies demonstrated generalized spike-and-wave discharges suggestive of epileptic activity. This discrepancy between clinical presentation and EEG findings underscores the importance of vigilance in reassessing the diagnosis and considering alternative etiologies [12]. EEG remains a cornerstone in the diagnostic workup of seizure disorders, but its limitations should be acknowledged, particularly in cases with atypical presentations [13]. The diagnosis of myoclonic epilepsy was ultimately established based on the presence of generalized spike-and-wave discharges on EEG, coupled with the patient's clinical history of myoclonic jerks. Myoclonic epilepsy encompasses a spectrum of disorders characterized by myoclonic jerks, often associated with generalized epileptiform discharges on EEG [14]. The identification of specific electrographic patterns on EEG aids in distinguishing between different epilepsy syndromes and guiding treatment decisions [15].

The initiation of anti-epileptic medication (valproate) resulted in a significant reduction in the frequency of the patient's episodes, further supporting the diagnosis of myoclonic epilepsy. Treatment of epilepsy aims to achieve seizure control while minimizing adverse effects and improving quality of life [16]. Valproate is commonly used in the management of myoclonic epilepsy due to its broad-spectrum anti-epileptic effects [17].

## Conclusions

This case exemplifies the diagnostic challenges inherent in distinguishing between psychogenic nonepileptic seizures and epileptic seizures, particularly when their clinical presentations are not clear-cut and EEG studies are normal. Despite initial assessments suggesting PNES, further investigation, including repeat EEG studies, led to the correct diagnosis of myoclonic epilepsy. This case highlights the importance of a comprehensive evaluation, incorporating clinical, EEG, and imaging findings, in arriving at an accurate diagnosis and implementing appropriate management strategies. A collaboration between neurologists, epileptologists, and psychologists is crucial in navigating such cases, ensuring optimal patient care and outcomes. Additionally, the successful initiation of anti-epileptic medication resulting in a reduction of seizure frequency underscores the importance of timely and tailored treatment interventions in epilepsy management. This case emphasizes the significance of ongoing vigilance and a multidisciplinary approach in evaluating and managing seizure disorders.
